# New clinical opportunities of low-field MRI: heart, lung, body, and musculoskeletal

**DOI:** 10.1007/s10334-023-01123-w

**Published:** 2023-10-30

**Authors:** Ye Tian, Krishna S. Nayak

**Affiliations:** Ming Hsieh Department of Electrical and Computer Engineering, Viterbi School of Engineering, University of Southern California, 3740 McClintock Ave, EEB 406, Los Angeles, CA 90089-2564 USA

**Keywords:** Low-field MRI, Body MRI, Lung, Cardiac, Abdominal, Musculoskeletal, Body composition

## Abstract

Contemporary whole-body low-field MRI scanners (< 1 T) present new and exciting opportunities for improved body imaging. The fundamental reason is that the reduced off-resonance and reduced SAR provide substantially increased flexibility in the design of MRI pulse sequences. Promising body applications include lung parenchyma imaging, imaging adjacent to metallic implants, cardiac imaging, and dynamic imaging in general. The lower cost of such systems may make MRI favorable for screening high-risk populations and population health research, and the more open configurations allowed may prove favorable for obese subjects and for pregnant women. This article summarizes promising body applications for contemporary whole-body low-field MRI systems, with a focus on new platforms developed within the past 5 years. This is an active area of research, and one can expect many improvements as MRI physicists fully explore the landscape of pulse sequences that are feasible, and as clinicians apply these to patient populations.

## Introduction

Clinical MRI hardware has trended towards better gradient performance (amplitude and slew rate), more sophisticated pulse sequences and reconstruction, and higher B0 field strengths (3 T, 7 T, and beyond). The first two developments have enhanced almost all MRI applications. The trend towards higher B0 field strength has primarily benefitted high-resolution static imaging of the brain, spine, and musculoskeletal system (in the absence of metallic hardware), which are the dominant use cases for MRI in radiology today, accounting for > 70% of clinical volume. The use of higher field strengths poses several challenges for body imaging, which is the topic of this review article. Major challenges include main field (B0) inhomogeneity, RF transmit (B1+) inhomogeneity, and high specific absorption rate (SAR) [1]. These limit the ability to image near tissue-air boundaries [2], image tissue adjacent to metallic implants [3, 4], optimize image contrast, and image deep organs [5].

There has been substantial recent interest in low-field MRI systems that are paired with high-performance shielded gradients and modern consoles, specifically for body and interventional imaging. Manufacturing low-field MRI systems with modern techniques can have several advantages, such as improved B0 homogeneity, favorable changes in NMR relaxation parameters (shorter T1, longer T2 and T2*), relaxed SAR constraints, reduced acoustic noise, reduced safety concerns, and reduced manufacturing cost and total cost of ownership [6–10]. A landmark paper in 2019 by Campbell-Washburn et al. [11] introduced the opportunities for improved imaging with contemporary low-field whole-body MRI. The work was based on a commercial 1.5 T “ramped down” to 0.55 T. A few years later, Siemens announced a commercial 0.55 T scanner Free.Max [12]. Guenthner et al. described opportunities availed at 0.75 T, based on a “ramped down” Philips 3 T scanner [13]. MR-Linac 0.35 T scanners [14] designed for MR-guided radiotherapy have also been leveraged to explore opportunities for general-purpose body imaging.

Diagnostic body imaging is a broad area that includes applications to the heart, lung, abdominal organs, and joints. This term notably excludes the brain and excludes interventional applications. Imaging the trunk area has several unique challenges as artifacts can arise [15] because of cardiac and respiratory motion, susceptibility gradient between lung and tissue that results in an inhomogeneous B0 field, and the proximity of fat and lean tissues that requires fat suppression or fat/water separation. Implanted metallic hardware, such as orthopedic implants, sternal wires, and cardiac devices introduce even more significant off-resonance effects [16]. Dynamic imaging of the cardiopulmonary systems or musculoskeletal (MSK) systems may be constrained by SAR limitations [17, 18], and off resonance and short T2/T2* may limit the use of efficient long readouts [19]. These issues are all mitigated at a lower field strength, providing opportunities for improved body imaging [11].

This article reviews the application of contemporary whole-body low-field MRI systems to body imaging. Table 1 contains a high-level summary of the advantages, consequences, and newly enabled applications of this configuration. We focus on recent developments, mostly within the past five years, and do not discuss historical work that is covered elsewhere in this special issue [20]. We do not cover interventional applications, as they are described by several other recent review papers [11, 21, 22]. We do not cover point-of-care body MRI systems [23], such as the prostate [24], or liver [25, 26] scanners, because these systems operate at a much lower field strength (< 0.1 T) and are designed for a single or a few applications, and are, therefore, not suitable for general-purpose body imaging. The results discussed in this paper come primarily from whole-body systems such as the 0.35 T (Viewray) [14], 0.55 T (“ramped down” Siemens Aera [11] and Siemens Free.Max [12]), and 0.75 T (“ramped down” Philips Achieva) [13], with specifications listed in Table 2.

**Table 1:** Advantages of low-field MRI systems

Properties of low-field systems	Imaging consequence	Applications impacted and example references
Reduced off-resonance (includes chemical shift and susceptibility): proportional to B0	Reduced bSSFP banding artifacts Reduced artifacts for long readouts (e.g., spiral and EPI) Reduced artifacts from metallic implants and instruments Smaller chemical shift	bSSFP cardiac [33, 35, 41] Imaging near metal (e.g., orthopedic implants) [86, 89, 90] Imaging near air spaces (lung, bowel, airway) [57–59, 63–66, 69, 92] Water/fat separation [106]
Reduced SAR: proportional to B0^2^	Allowing higher flip angles (imaging and refocusing) Allowing higher bandwidth pulses (higher peak B1+)	bSSFP imaging where a high flip angle is contrast optimal [33] Simultaneous multi-slice imaging [40, 50]
Favorable relaxation properties: shorter T1 and longer T2/T2*	Stronger signal for certain sequences (e.g., bSSFP and GRE), partially compensates for the reduced SNR due to polarization	Lung parenchyma imaging [55] Improving SNR for fast gradient echo pulse sequences [41]
Enabling larger bore size	Improved patient comfort Ability to image obese subjects Ability to achieve different in-bore postures (e.g. side laying) Space to perform movements during imaging	All existing imaging [7, 92]
Reduced acoustic noise	Improved patient comfort	All existing imaging [7, 9]
Reduced cost (total cost of ownership)	Improved access	All existing imaging [8]

**Table 2:** Contemporary whole-body low field (< 1 T) systems

	MR-Linac	“ramped down” Aera 1.5 T	Free.Max	“ramped down” Achieva 3 T
Vendor	Viewray	Siemens	Siemens	Philips
Field strength	0.35 T	0.55 T	0.55 T	0.75 T
Maximum slew rate	200 T/m/s	200 T/m/s	40 T/m/s	200 T/m/s or 100 T/m/s
Maximum gradient amplitude	18 mT/m	45 mT/m	25 mT/m	40 mT/m or 80 mT/m
Bore size	70 cm	70 cm	80 cm	60 cm

## Cardiac imaging

Cardiac MRI is one of the major applications that benefit from high-performance low-field systems [11, 27–30]. Advantages include an improved safety profile due to lower SAR and improved device compatibility [31], improved ECG gating due to the reduced magnetohydrodynamic effect [32], and reduced off-resonance-related artifacts particularly for sequences like balanced steady-state free precession (bSSFP) [11].

**Fig. 1:**
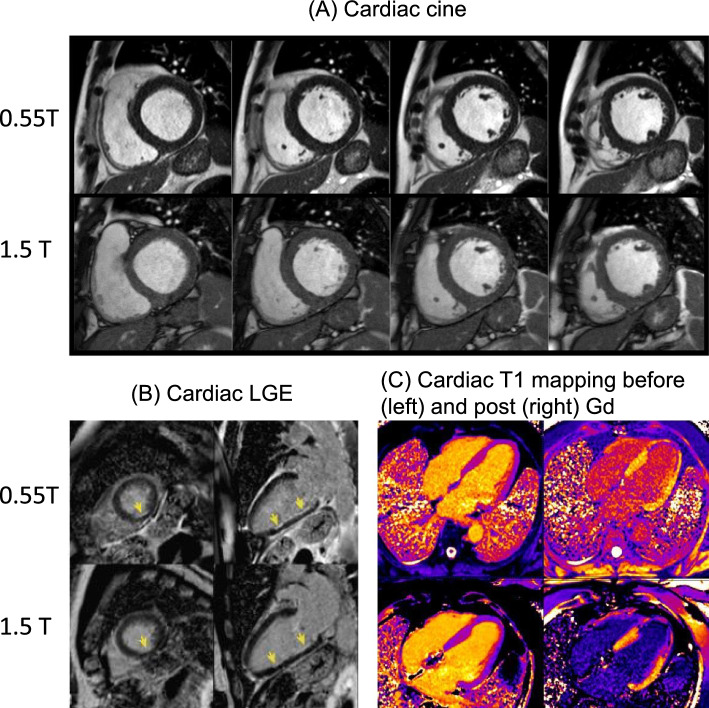
Comparison of cardiac MRI between 0.55 T and 1.5 T. **A** cardiac cine, reproduced from Bandettini et al. [35] **B** cardiac LGE, reproduced from Bandettini et al. [38] **C** cardiac T1 mapping, reproduced from [37]. All illustrated cardiac applications on 0.55 T show comparable diagnostic values as 1.5 T

An important first question to ask is whether low-field systems can achieve equivalent diagnostic performance compared to traditional field strengths, with particular attention to the reduced SNR due to polarization. Researchers have compared low-field cardiovascular magnetic resonance (CMR) with 1.5 T CMR for diagnostic performance. Rashid et al. [33] compared cine CMR at 0.35 T with 1.5 T, and found that 1.5 T cine provided higher SNR and blood-myocardium CNR, however, expert qualitative scores for high flip angle (≥ 90°) 0.35 T cine was comparable to 1.5 T cine. Varghese et al. [34] compared cardiac function, blood flow, and myocardial tissue relaxation times between 0.35 T and 1.5 T. The study reported lower SNR at 0.35 T, but ventricular volumes, ejection fraction, peak velocity, and stroke volumes were all comparable between 0.35 T and 1.5 T or 3 T. At 0.55 T, a major effort led by intramural researchers at the National Institutes of Health (NIH) compared different cardiac sequences between 0.55 T and 1.5 T, and these included cardiac cine for functional analysis [35], late gadolinium enhancement [36], and T1 mapping [37]. Bandettini et al. [35] compared cardiac cine between 0.55 T and 1.5 T, and found that ventricular volumes, ejection fraction, left ventricular mass, and diagnostic performance for the detection of regional wall motion abnormality were comparable. Their study focused on myocardial infarction quantification by late gadolinium enhancement imaging [38] found that both myocardial infarction mass and percentage of infarction were comparable between 0.55 T and 1.5 T. Mancini et al. [37] compared cardiac T1 mapping between 0.55 T and 1.5 T found that while T1 values are significantly lower at 0.55 T, as expected, extracellular volume fraction estimated from pre/post gadolinium T1 maps at the two field strengths showed strong agreement. Figure 1 shows representative comparisons between prototype Aera 0.55 T and 1.5 T MRI for (A) cardiac cine, (B) cardiac late gadolinium enhancement (LGE), and (C) T1 mapping. Varghese et al. have evaluated a comprehensive cardiac MRI protocol on the commercial Free.Max 0.55 T system [39], demonstrating reasonable image quality for cardiovascular structure, function, flow, and LGE assessments. Although the aforementioned studies are relatively small scale (< 500 subjects), they have demonstrated diagnostic cardiac imaging at 0.35 T and 0.55 T.

**Fig. 2:**
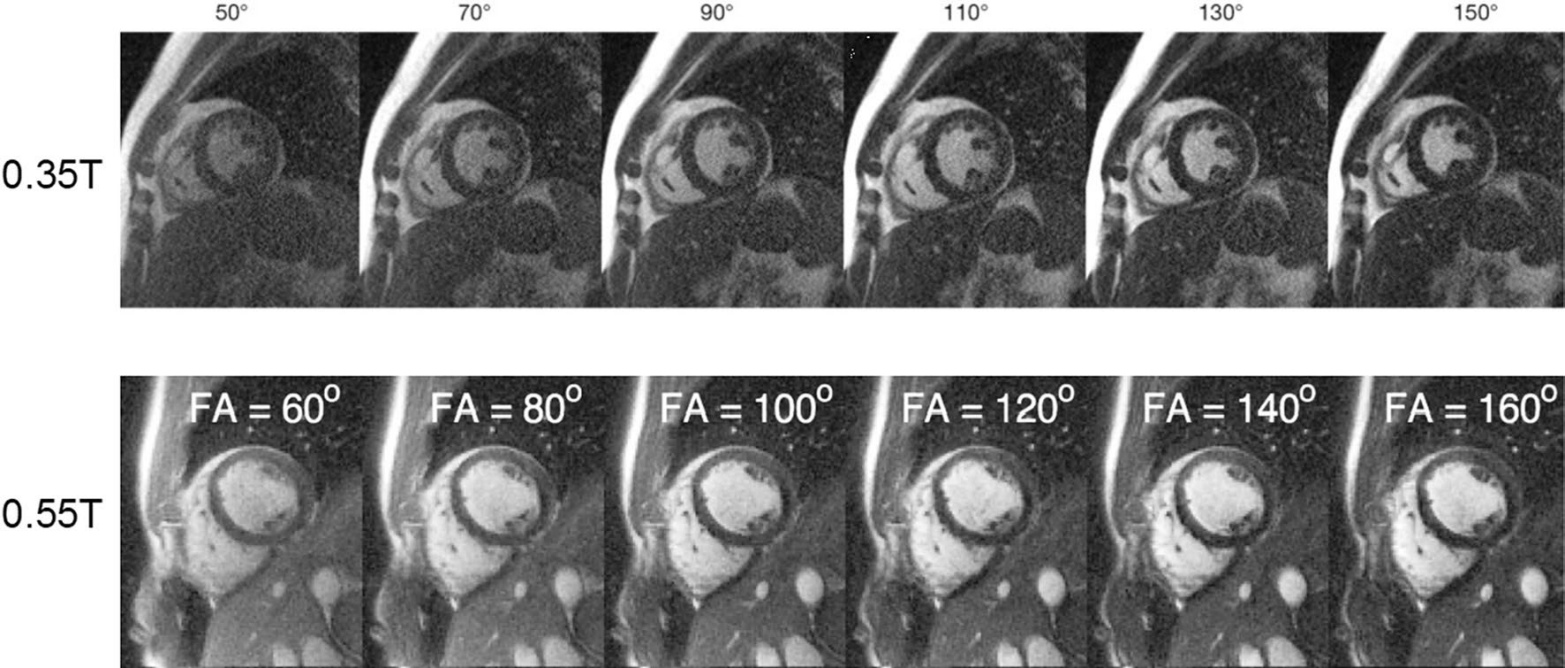
Blood-myocardium contrast changes with a flip angle. The apparent contrast between the blood and the myocardium increases as the bSSFP flip angle increases and peaks at flip angle = 130° at 035 T and at flip angle = 160° at 0.55 T. Note that the blood signal is not in a steady state due to the constant inflow in the ventricular, which increases the signal intensity. This resulted in a higher contrast-optimal flip angle in the experiment than in the steady-state Bloch simulation. A high flip angle can be applied within a reasonable TR without exceeding the SAR limitation on low-field systems. At 1.5 T or 3 T, applying a high flip angle may prolong the TR, which can introduce more banding artifacts for bSSFP cine. 0.35 T images are reproduced from Rashid et al. [33] and the 0.55 T images are reproduced from Tian et al. [40]

Researchers have also studied protocol improvements to compensate for the reduced SNR due to polarization at 0.35 T [33] and 0.55 T [40], which suggest the use of higher flip angles and longer readouts. Figure 2 shows an example of bSSFP cardiac cine images acquired with different flip angles at 0.35 T and 0.55 T, demonstrating a high flip angle (usually ≥ 90°) yields the optimal blood-myocardium contract. The use of a high flip angle at 1.5 T or 3 T is usually problematic due to the SAR constraints. Low-field systems also support contrast-optimal flip angle for simultaneous multi-slice (SMS) bSSFP, as reported by Tian et al. [40]. Restivo et al. [41] demonstrated that with an SNR efficient sampling trajectories such as spiral in–out readout, there is a 79% increase in the SNR when compared with Cartesian readout, and the SNR reduction when compared with 1.5 T Cartesian is 48% in the blood and 31% in the myocardium. These SNR-efficient techniques such as SMS and long readout may be problematic at higher field strength due to increased SAR and off-resonance artifacts. On the 0.75 T system, Peereboom et al. [42] measured T1, T2, and T2* in the myocardium, and demonstrated that by leveraging these physical prosperities in the pulse sequence design accurate myocardial spectroscopy measurement can be achieved at 0.75 T.

Real-time cardiac imaging is promising at low field since the ability to use long readouts (such as spiral or echo-planar [41]) dramatically improves scan efficiency. Real-time imaging generally provides simplified and patient-friendly scanning because it does not rely on breath-holds or ECG gating. However, the use of non-Cartesian trajectories and high undersampling factors can limit the speed of online reconstruction. In cases where low-latency is needed, through-time GRAPPA [43, 44] and machine learning-based reconstructions [45] have been shown to provide reconstruction with ≤ 200 ms latency [46]. At low field, Fyrdahl et al. [47] demonstrated the feasibility of through-time GRAPPA for real-time cardiac MRI on the Free.Max 0.55 T. On the same system, Hamilton et al. [48] developed a deep-learning reconstruction for real-time cardiac MRI. Real-time cardiac MRI capability is greatly improved by leveraging the ability to use longer readouts, flexibility of trajectory design, and relaxed SAR constraints. These have been demonstrated on the prototype Aera 0.55 T system. Wang et al. [49] developed a spiral in–out sequence for real-time cardiac cine, which provides improved scan efficiency and achieves TE approximately equal to TR/2. Yagiz et al. [50] developed an SMS bSSFP technique at 0.55 T to achieve real-time simultaneous multiple-slice coverage, enabling more comprehensive real-time cardiac function assessment.

To date, several pilot studies have demonstrated the feasibility of low-field CMR for cardiac function, tissue characterization, flow measurements, and LGE imaging. Some functional assessments may be worse at low field, such as those based on feature tracking [51] and tagging [52], due to the need for high SNR and/or long myocardial T1 for tag persistence. Myocardial first-pass perfusion can benefit from expanded coverage and a finer spatial resolution, that low-field systems may be able to provide [53]. CMR at low field may also provide reduced safety concerns and reduced imaging artifacts for patients with cardiac implanted devices [54], providing improved access and screening for such patients. Clinical evaluation of real-time CMR is still needed to assess its robustness and workflow improvements. The focus should be on patients with arrhythmia or those who experience difficulties in maintaining breath-holding during the imaging process.

## Lung imaging

Lung imaging is an application that is currently dominated by computed tomography (CT), where low-field MRI could provide a breakthrough [11]. CT provides excellent spatial resolution in a short scan time, but requires exposure to ionizing radiation, and has difficulty characterizing tissues (e.g., differentiating benign from malignant lung nodules). The radiation dose is a constraint when deciding the frequency of monitoring, use in children, and feasibility of dynamic assessments. MRI, being a radiation-free modality that is excellent for tissue characterization, has the potential to alleviate these constraints. Lung MRI at conventional field strengths is limited by the extremely short T2* in parenchyma (due to the numerous air-tissue interfaces from alveoli). This is a much relaxed constraint at low field and can greatly improve structural imaging and functional assessments of the lung.

**Fig. 3:**
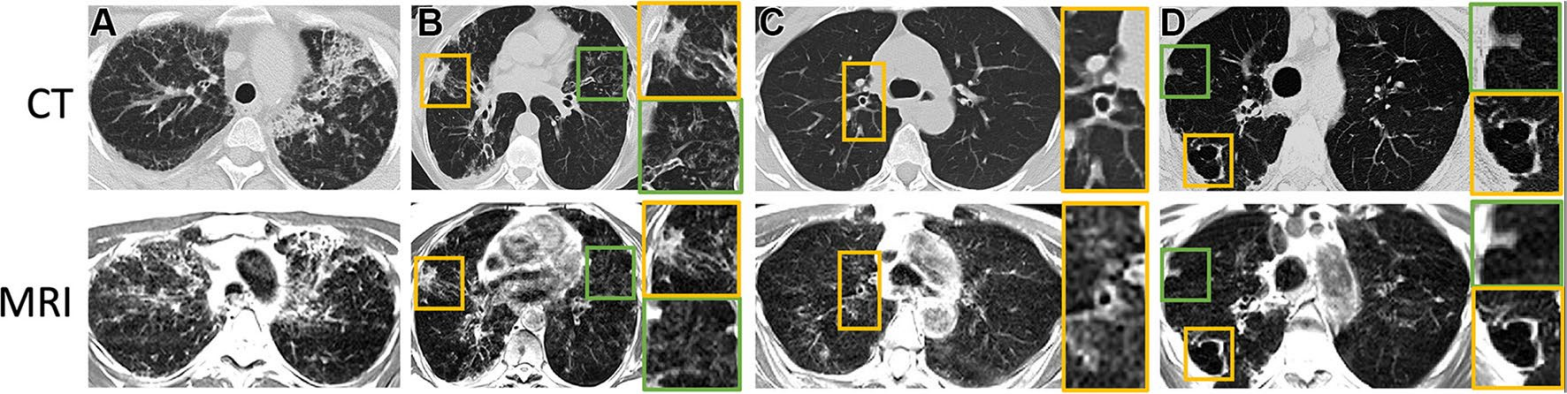
Axial CT (reformatted to 0.8 × 0.8 × 6 mm) and T2-weighted MR (reformatted to 1.1 × 1.1 × 6 mm) images show **A** honeycombing, interstitial thickening, and fibrotic changes in a 35-year-old woman; **B** consolidative opacities in a 70-year-old woman; **C** bronchial wall thickening with bronchiectasis in a 58-year-old woman; and **D** a cavitary lesion in a 70-year-old woman. **B**–**D** Green and yellow boxes on CT and MR images denote the area of interest around the particular finding described, with the corresponding box bordered in that color to the right of the respective image showing an enlarged view of the area. Image is reproduced from Campbell-Washburn et al. [57]

At low field, lung parenchyma T2/T2* values are greatly prolonged, providing high SNR efficiency. Li et al. [55] jointly measured the lung parenchyma T2/T2* with an echo-shifted multi-echo spin echo pulse sequence, determining the normative values of lung parenchyma are T2 = 68.6 ms and T2* = 8.2 ms. Notably, the T2* is 5–10 times larger than at 1.5 T (2.11 ms) or 3 T (0.74 ms) [56]. This greatly improves the capability of performing lung imaging at low field strengths, and many opportunities are emerging. Campbell-Washburn et al. [57] demonstrated diagnostic image quality with a T2-weighted spin echo sequence in a small cohort of patients (*N* = 24) with various lung conditions, with representative examples shown in Fig. 3. T2-weighted sequences at 0.55 T provide important structural assessments of many lung conditions. Combined with a diffusion weighted imaging, differentiation of benign and malignant lung nodules was also possible [58]. T2-weighted structural images were also used to assess pulmonary ground glass and fibrosis-like opacities associated with COVID-19 [59–61]. Hinsen et al. [62] compared lung nodule detection between 0.55 T MRI and CT. In 964 total nodules of 46 patients, MRI had 100% accuracy in detecting nodules of size ≥ 6 mm, 80% (159/200) for those ≥ 4 and < 6 mm, and 23% (147/638) for those < 4 mm.

Ultra-short TE (UTE) imaging, by using extremely short excitation pulses and center-out readouts, can overcome the short T2* limitation at high field strengths. UTE has been used with great success, however, is limited by short readout time (< 2 ms) and poor scan efficiency. At low field, UTE pulse sequences can be combined with long spiral readout to achieve improved scan efficiency. Bhattacharya et al. [63] studied the use of breath-held spiral UTE to evaluate regional lung ventilation based on the T1-shortening effect of inhaled 100% O_2_ (compared to room air). By using a 7-ms spiral readout, the sequence achieved 3.5 × 3.5 × 10 mm^3^ whole-lung coverage in a short 9-s breath-hold. This combined with a T2-weighted BLADE pulse sequence enabled joint assessment of lung function and structure in patients with lymphangioleiomyomatosis (LAM) [64]. Javed et al. [65] have demonstrated a free-breathing UTE spiral pulse sequence for 3D lung imaging, achieving 1.75 mm isotropic resolution whole-lung imaging in 8 min. This pulse sequence was further used to study the lung water density, where it was shown that with a lower spatial resolution at 3.5 mm isotropic and 1° flip angle it was sensitive enough to capture lung water redistribution under gravity [66]. bSSFP with an extremely short TR (~ 2 ms) can provide high-quality lung structure imaging at 1.5 T [67, 68]. This approach has recently been shown to achieve sub-millimeter lung imaging at 0.55 T [69].

Hyperpolarized ^3^He imaging is also promising at the low field since the polarization is not dependent on field strength, the increased T2 and T2*, and reduced susceptibility gradients enabled more flexible and efficient redouts. Though there are no studies investigating hyperpolarized lung imaging with contemporary low-field systems, some other works are worth mentioning. Komlosi et al. [70] studied the SNR, T2, and T2* with hyperpolarized ^3^He at different field strengths (0.43 T, 0.79 T, and 1.5 T), finding the SNR weakly depended on field strength, however, there is a large increment in T2 and T2* values at lower field strengths, as expected. An earlier work by Salerno et al. [71] reported a threefold increment in hyperpolarized ^3^He T2* value at 0.54 T compared with 1.5 T, and reduced spiral blurring induced by susceptibility effects.

## Abdominal imaging

Abdominal imaging is challenging because of the large field-of-view (FOV) requirement, inhomogeneous B0 field due in part to gas in the gastrointestinal (GI) tract, and complex motion from breathing and gastrointestinal movements. Abdominal imaging can benefit from low-field systems, due to the improved B0 field homogeneity, reduced artifact near lung air or GI gas, and reduced artifacts near metallic implants [72]. Low-field systems may also be made with larger bore size, enabling imaging for obese patients [73]. Relaxed constraints in pulse sequence design such as enabling large FOV bSSFP, reduced SAR, and enabling longer readout also benefit dynamic imaging, for example, studies for GI movements or motion-resolved abdominal imaging [74].

**Fig. 4:**
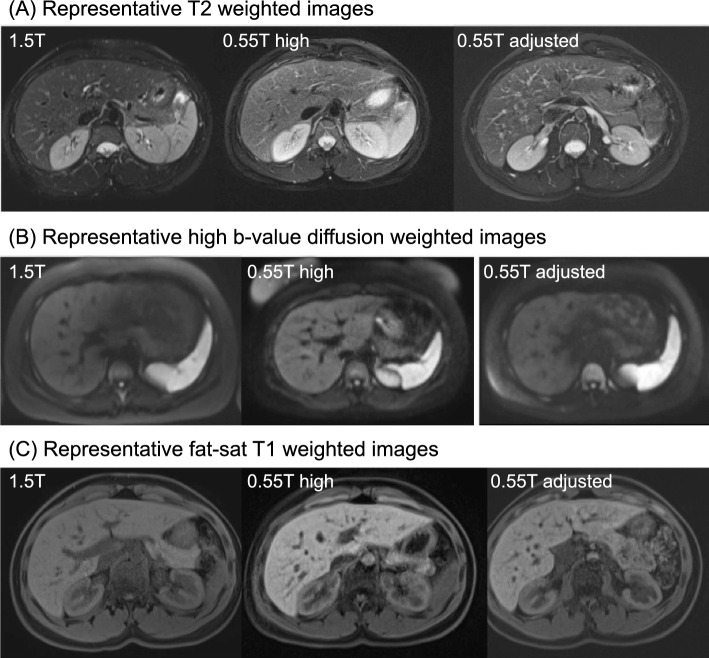
Representative liver images at 1.5 T, 0.55 T with a high gradient, and 0.55 T with an adjusted gradient. **A** T2 weighted images **B** high b-value diffusion-weighted images **C** T1 weighted fast spin echo images. Each row shows images acquired at 1.5 T (left), 0.55 T with “ramped down” Aera gradient performance (middle), and 0.55 T with gradient performance adjusted to the same as Free.Max

Despite the promises of low-field abdominal imaging, there is little published work in this area. Chandarana et al. [75] developed a protocol for abdominal imaging at 0.55 T. In 10 healthy volunteers, the group demonstrated diagnostic quality images with fat-saturated T2 weighting, diffusion weighting, and Dixon T1 weighting in a total acquisition time of 10 min. Representative images from this protocol are shown in Fig. 4. Ramachandran et al. [76] compared 0.55 T MRI and 1.5 T MRI of the abdomen in 15 healthy volunteers, finding that the 0.55 T achieved an acceptable image with a prolonged scan time. Issues that warrant further investigation are low SNR, insufficient fat suppression, and aliasing due to undersampling. Liu et al. [77] have studied the use of Rosette MR fingerprinting for quantifying T1, T2, and water-fat separation in the liver at 0.55 T. The use of Rosette trajectory provides multiple echoes for water-fat separation, and the noise-resilient fingerprinting with longer readouts can partially overcome the low SNR issue at low field. Another fingerprinting work performed at 0.75 T also targeted at water/fat-separated parameter mapping in the abdomen but with a spiral multi-echo acquisition [78]. Future studies are needed to investigate the diagnostic performance of low-field MRI for specific diseases and to develop methods to suppress confounding artifacts. Opportunities exist in exploring applications that benefit from reduced off-resonance artifacts around gas and in a larger FOV.

## Musculoskeletal imaging

Contemporary low-field systems may provide diagnostic images for most MSK applications. Khodarahmi et al. [79] reviewed MSK applications at low field and posed challenges and opportunities in this area. The low SNR at low field is the largest challenge for general MSK applications since MSK applications generally require fine spatial resolution and high contrast to identify injuries or degenerative disorders. Solutions to these issues include the use of longer scan time and AI-based denoising. Another major challenge in MSK at the low field is water-fat separation. As the water fat chemical shift frequency is smaller at lower field strength, water or fat suppression preparation pulses are prolonged and are not as effective [80]. Short tau inversion recovery (STIR) can be applied to effectively suppress the fat signal at low field, albeit with reduced flexibility in image contrast [81]. Despite the challenges, low-field systems may have several advantages over higher-field strength systems in imaging near metallic implants and dynamic imaging of the MSK system. These advantages mainly come from the reduced susceptibility effects and improved dynamic imaging capability.

A few early and recent cross-field strength comparison studies have demonstrated the diagnostic capability of several MSK applications at low field. In an early study done in 1996, Rutt et al. [82] demonstrated no diagnostic difference between early 0.5 T and 1.5 T scanners for anterior cruciate ligament tears in the knee. A recent study compared lumbar spine images in healthy volunteers acquired at both 0.55 T and 1.5 T [83]. The study revealed that the image quality at 0.55 T is perceived to be lower than 1.5 T. The 0.55 T images underwent denoising using a machine learning tool provided by the vendor (Deep Resolve Gain/Sharp, Siemens Healthcare, Erlangen, Germany), resulting in improved image quality. Although the denoised 0.55 T images were still rated lower than 1.5 T images, they were in the good to perfect range and were acquired with a reduced scan time. With the same deep learning tool, Schmidt et al. [84] demonstrated that deep learning enhanced MRI images at 0.55 T could achieve equivalent diagnostic accuracy compared to 1.5 T MRI for knee structural and pathological diagnosis.

**Fig. 5:**
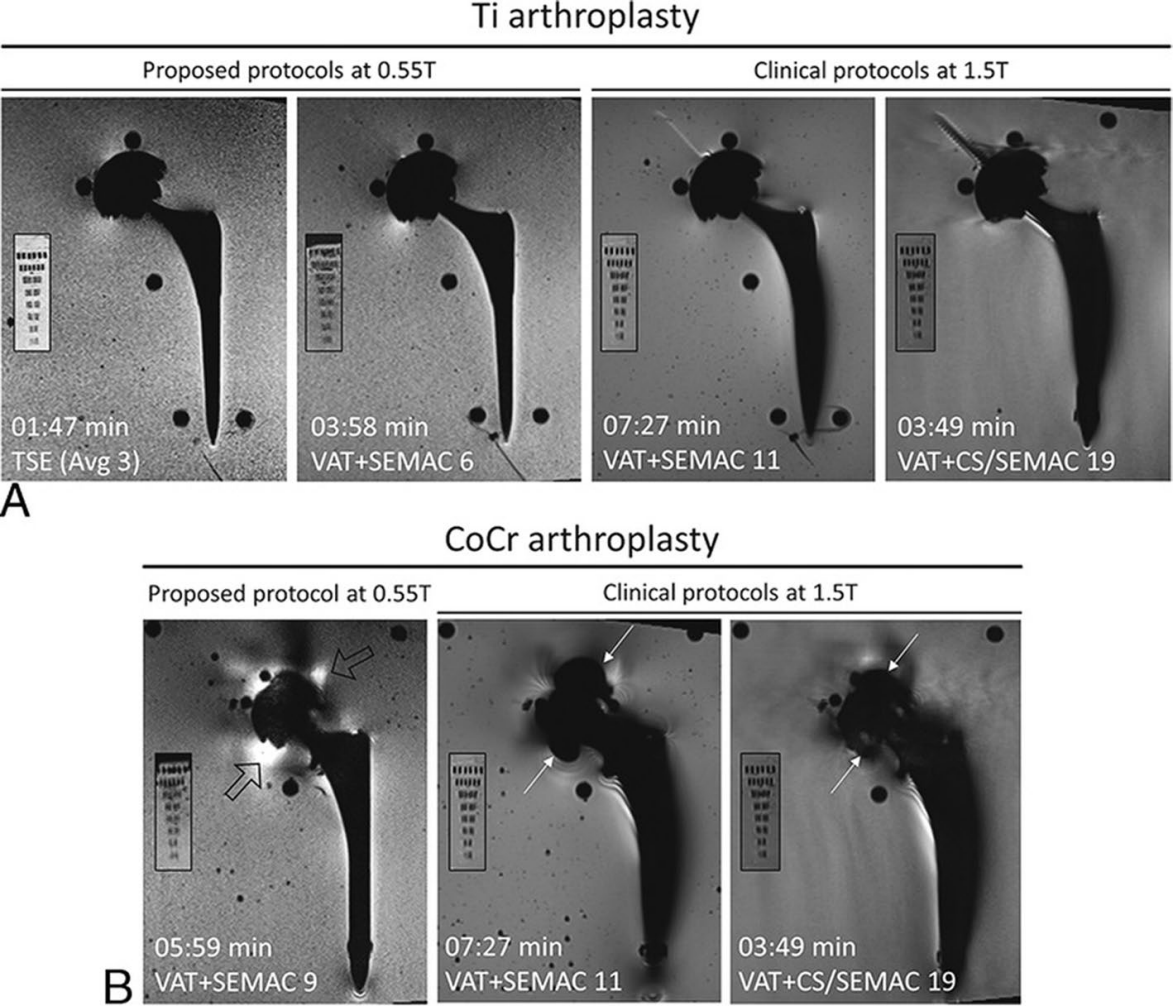
Comparison of 0.55 T optimized pulse protocols with clinical 1.5 T View Angle Tilting (VAT) + Slice Encoding for Metal Artifact Correction (SEMAC) and VAT + CS/SEMAC pulse sequences for Ti (**A**) and CoCr (**B**) implants. Smaller panels show the resolution phantom in each case. Metal artifacts are significantly reduced primarily surrounding the femoral stem for both implant types. Areas of signal loss around the CoCr acetabular cup at 1.5 T (white arrows) are replaced by smaller areas of signal pileup at 0.55 T (hollow arrows). Image is reproduced from Khodarahmi et al. [89]

Low-field systems have the potential to significantly enhance the ability of imaging around metallic implants. Depending on the material, size, shape, and orientation, metallic implants create different levels of imaging artifacts at different field strengths [16, 85], due to the large susceptibility gradients generated by metal. Certain materials have consistent artifacts across field strengths [86, 87]; however, most materials produce artifacts that scale with field strength. Consequently, imaging tissues adjacent to metallic implants becomes more challenging at higher field strengths [88]. At lower field strengths, susceptibility effects are much smaller, such that the distortion, signal void, and signal pile-up effects are much reduced. Khodaremi et al. [89] have compared hip implant phantom imaging using SEMAC between 0.55 and 1.5 T and found that with an optimized protocol the 0.55 T MRI has a 45–64% smaller artifact size compared with 1.5 T MRI, with only a minor SNR penalty at 17–28%. Representative hip implant phantom images acquired at 0.55 T and 1.5 T are shown in Fig. 5. Keskin et al. [90] studied the use of gradient sequences to obtain high-resolution images near metallic implants, which is usually not feasible at higher field strengths.

Dynamic imaging of the MSK systems is also promising in contemporary low-field systems. These systems allow for longer readouts, higher flip angle, and bSSFP imaging. Lim et al. [91] studied the use of real-time bSSFP sequence for speech imaging at 0.55 T, reporting superior image quality than spoiled gradient echo (GRE) sequence at the same and higher field strengths. The feasibility of using real-time MRI for assessing wrist movement instability with a high spatial resolution was also demonstrated at the ramped-down Aera 0.55 T system [92].

Several low-field open-bore systems also have a role in clinical MSK imaging, such as the G-scan Brio 0.25 T (Esaote) and the MrOpen 0.5 T (Paramed). These systems are specially designed to support imaging at multiple body positions, including but not limited to supine, sitting, and standing, each poses a different weight loading (e.g., in the knee or the spine). Comparison of MSK under different realistic weight loading conditions can better elucidate pathology and the underlying reason for symptoms including pain. Alessandra et al. [93] reported the use of a 0.25 T system for assessing lumbar spine instability over 10 years on 4305 patients. When imaging at both supine and standing positions, they were able to assess disc diseases that were undetected when imaging in the supine position alone. Morphological changes in the spine under different loading conditions were also reported by Nordberg et al. [94] with the same scanner. At 0.5 T, Aarvold et al. [95] reported femoral head deformity under weight bearing in children with Legg-Calve-Perthes disease. Pai et al. [96] studied muscle morphology changes in the thorax with the level and posture of healthy volunteers. Charest-Morin et al. [97] studied dynamic morphometric changes in the spine between supine and standing positions in patients with degenerative spondylolisthesis.

Small bore low-field “extremity” MRI systems can be made with more compact sizes since only one limb needs to fit, and a dedicated scanner shape and coil for the targeted region of interest can be designed [98, 99]. A few studies have evaluated the diagnostic performance of the E-scan 0.2 T shoulder system, generally supporting its usefulness in the diagnosis of conditions such as rotator cuff and labral lesions [100, 101]. Wrist and finger joints assessments using the 0.2 T Artsocan system were also studied [102–104], supporting the cost-effectiveness and accuracy of the diagnosis.

## Body composition

Whole body composition or organ-specific body composition measurements are valuable biomarkers of obesity, metabolic disease, muscle disorders, and diagnostic references. This usually requires water/fat-separated imaging to quantify the fat–water proton density ratio. Low-field systems may provide improved body composition measurements by enabling larger bore size, reducing acoustic noise, and reducing scan time by leveraging time-efficient readouts. At low field strength, there are unique technique challenges for water/fat separation or spectrum excitation. ^1^H in water and fat experience different chemical shifts. The difference between water (single-peak) and the dominant peak in lipids is approximately 3.5 parts per million. The difference in off-resonance frequencies (Δf) is directly proportional to the field strength. The smaller Δf at low field makes it less practical to apply frequency selective pulses, since these pulses would require longer duration (roughly 1–2 times the reciprocal of ∆f, e.g., 12–24 ms at 0.5 T). Methods that separate water and fat components, such as the multiple echo Dixon method [105], can be used to achieve water or fat suppression effectively. Nayak et al. [106] recently optimized a 2-point Dixon protocol for body compensation profiling at 0.55 T. Several works have targeted more sophisticated optimization of Dixon methods at low field, including a 3-point Dixon at 0.75 T [107], and a combination with non-Cartesian trajectories to achieve improved scan efficiency [108] or motion robustness [109]. Tian et al. have demonstrated a bSSFP spiral out-in–out-in sequence for real-time water/fat separation [110]. The use of bSSFP pulse sequence and spiral readout improves the SNR efficiency, allowing real-time imaging with sufficient SNR for water/fat separation. However, the drawback of bSSFP is its susceptibility to banding artifacts and signal void, which can pose challenges for fat fraction quantification.

Zi et al. [111] demonstrated a frequency offset method applied to adiabatic spectrally selective inversion pulse for effective fat suppression in abdominal imaging. Instead of designing an effective fat suppression pulse, the work exploits a short preparation pulse but sweeps a range of RF frequencies and uses the frequency response to perform effective water and fat separation. Note that the scan time is prolonged due to the frequency sweeping, however, the redundancy of data may allow for more sparse sampling with constrained reconstruction.

## Technical challenges and opportunities

There are known issues at low field, namely the concomitant field effects and the lower SNR due to polarization. Lee et al. [112] have addressed the concomitant field problems for GRE-based spiral sequences in the MaxGIRF framework. MaxGIRF uses a model-based reconstruction and can simultaneously correct the off-resonance and concomitant fields blurring, but a separate measurement of the off-resonance map is needed. The group is also extending the work to include gradient nonlinearities in the reconstruction modeling [113]. Wang et al. [114] have developed a modification to the spiral-ring spin echo pulse sequences to compensate for concomitant field effects. This method nulls the concomitant field at the echo time by modifying the gradient, and when combined with reconstruction-based concomitant field correction and off-resonance correction, imaging blurring in spiral imaging is substantially reduced. With a similar approach, Ramasawmy et al. [115] compensated concomitant field for a turbo spin echo spiral in–out sequence.

Pilot tone [116] is a promising technique to capture respiratory and cardiac physiological signals without the need of external device or image navigator, by incorporating the motion signal into the acquired data, just outside of the imaging bandwidth. Pilot tone is applicable at all field strengths, and there are relatively minor (but non-trivial) technical challenges to getting consistent performance across field strengths due to the different operating frequencies [117]. Solomon et al. [118] demonstrated the feasibility of pilot tone on an Aera 0.55 T scanner, capturing a good quality respiratory signal that could be used to guide image reconstruction. Obtaining a reliable cardiac signal using pilot tone at low field remains an open challenge. As modern low field scanners are emerging recently, some of the product models have built-in pilot tone support such as Siemens Free.Max. Pilot tone may generally benefit free-breathing body imaging when respiratory motion needs to be resolved.

Application-specific coil design can greatly improve image SNR, parallel imaging performance, and sensitivity to the desired FOV. These have been demonstrated in the application of speech production [119] and in the knee [120] at 0.55 T. Coils designs are field-strength and frequency dependent, and improvements can be expected as more effort is dedicated to improved low-field body coils.

Powerful new tools based on machine learning are being used to enhance MRI capabilities [121, 122]. Example uses include image reconstruction and denoising [123–125], trajectory optimization [126–128], automated scan planning [129, 130], and automated analysis [131–133]. Although studies focusing on low-field strength (especially 0.1–1 T) are still rare, low-field MRI is expected to greatly benefit from these advances. Low-field MRI generally suffers from lower SNR due to lower equilibrium polarization, therefore we expect that denoising algorithms using machine learning may effectively mitigate this issue [134–136]. Machine learning can also contribute to sampling strategy optimization. Since low-field systems allow substantially greater flexibility in the design of sampling trajectories, longer readouts, and the utilization of high B1+ pulses, machine learning is expected to be a powerful aid in searching a broad parameter space to identify optimized solutions for specific applications. Machine learning also has a significant impact on scanning and analysis through automation. This potential reduction in the need for specialized personnel training can further lower operational costs associated with low-field systems, ultimately facilitating greater access to MRI services.

## Discussion

We have reviewed the body applications of contemporary low-field (< 1 T) MRI scanners. Most of the research has focused on the optimization of clinical protocols and demonstrating the feasibility or baseline diagnostic performance of these scanners, by using well-established 1.5 T MRI, 3 T MRI, or CT as a reference. To our knowledge, clinical non-inferiority studies have not been performed to date. These are expensive and difficult to perform, as they require very large numbers of subjects, and require each subject to undergo multiple exams.

Contemporary high-performance low-field MRI systems provide comparable and in many cases superior-performance body imaging compared to high-field counterparts. The reduced off-resonance and relaxed SAR provide substantially increased flexibility in the design of MRI pulse sequences. This is an active area of research, and one can expect many improvements in the coming years, as MRI physicists fully explore the broader range of pulse sequences that are feasible, and as clinicians apply these to patient populations.

Low-field outperforms conventional field strengths for some applications, notably lung imaging (because of the substantially longer T2*) and imaging immediately adjacent to metallic implants (because of the reduced susceptibility-induced off-resonance). We anticipate that low-field MRI may become the premier platform for these and other applications. Computed Tomography (CT) is the leading modality for diagnosis and evaluation of lung diseases. This is largely due to the limited imaging options in the lung, though the added radiation dose is of concern for certain patient groups such as young children or patients who need repeated scans. Low-field MRI has the potential to replace CT in certain applications, such as lung function evaluation [63], early detection of lung nodules [62], and evaluation of cystic fibrosis. Imaging around metallic implants is another application that low-field MRI has clear advantages over higher field strengths [89]. SNR remains as an issue due to the lower equilibrium polarization, and the need for very high spatial resolution. This is an area where novel multi-spectral imaging pulse sequences and reconstructions and/or machine learning may be helpful.

It remains an open question whether low-field MRI will gain broad traction prior to large-scale studies demonstrating clinical non-inferiority (compared to standard 1.5 T or 3 T MRI). Several small-scale studies have already demonstrated the diagnosis accuracy of low-field MRI, such as for cardiac [33–35, 38, 39], MSK [79], and abdominal [75] applications. These studies have also documented differences in image quality which trend as expected with field-strength differences that would be expected based on MRI physics.

Low-field MRI may enable novel evaluation of normal and abnormal body dynamics, such as joint movement [92, 137], bowel motility [110], swallowing/aspiration [139], and airway collapse [140]. These have been attempted at conventional field strengths but were difficult to make robust for widespread use. Dynamic MRI appears to be more robust at low-field, and this may provide a new suite of applications for MRI as a whole. Quantitative imaging at low field may provide new clinical insights because of relaxometry changes (shorter T1 and longer T2/T2*), Reference tissue quantification values need to be reestablished for each different field strength or different scanner [141, 142]. Another major promise of low-field MRI is improved accessibility. The reduced cost of ownership, reduced carbon footprint, and less liquid helium requirements could extend MRI services to areas that previously had limited access. It is often necessary to increase scan time to achieve acceptable image quality [39, 76, 79], which suggests the need to evaluate cost-effectiveness for low-field MRI systems. Improved patient comfort with reduced acoustic noise, larger bore size design, and improved safety, could bring MRI serve to people that was previously underserved [6, 7, 9, 143].
